# Effects of aflatoxin B1 on the submandibular salivary gland of albino rats and possible therapeutic potential of *Rosmarinus officinalis*: a light and electron microscopic study

**DOI:** 10.12688/f1000research.25196.1

**Published:** 2020-07-21

**Authors:** Bassant Ashraf, Dahlia Ghazy, Mohamed Shamel

**Affiliations:** 1 Department of Oral Biology, Faculty of Dentistry, October University for Modern Sciences and Arts, Giza, Egypt; 2 Department of Oral Biology, Faculty of Dentistry, Ain Shams University, Cairo, Egypt; 3 Department of Oral Biology, Faculty of Dentistry, The British University in Egypt, Shorouk city, Egypt

**Keywords:** Aflatoxin B1, Rosemary, Submandibular salivary gland, Antioxidant, electron microscope

## Abstract

**Background**: Aflatoxin B1 (AFB1), a highly toxic mycotoxin, is one of the contaminants of food items such as corn, rice, nuts, and flour. This study aimed to evaluate the effect of AFB1 on the histology and ultrastructure of the submandibular salivary glands (SMSG) of albino rats and examine the possible therapeutic effect of *Rosmarinus officinalis* extract.

**Methods**: This study used 21 adult male albino rats equally divided into three groups as follows: Group C (saline-treated control group); Group A **(**AFB1 treated group) subjected to intraperitoneal injection of AFB1 (2 mg/kg) once daily for four weeks; Group R (rosemary-treated group) subjected to AFB1 as in Group A followed by two weeks of intraperitoneal injection of *Rosmarinus officinalis* extract (400mg/kg) once daily. At the end of the experimental periods, SMSGs were excised and fixed for histological and ultrastructural examinations.

**Results:** SMSGs of the AFB1 group presented atrophied serous acini with numerous cytoplasmic vacuolations; their granular convoluted tubules, striated ducts and excretory ducts presented signs of degeneration in their cell lining with the presence of abundant cytoplasmic vacuolations. In addition, dilated blood vessels engorged with red blood cells were frequently seen. Ultrastructural findings of the AFB1 group showed some acinar cells with degenerated mitochondria presenting loss of cristae and vacuolations as well as irregular, shrunken nuclei with condensed chromatin. Dilated rough endoplasmic reticulum were observed in granular convoluted tubules and striated ducts. The glands of animals that received rosemary extract almost regained their normal architecture.

**Conclusions:** It can be concluded that rosemary extract has an ameliorative effect on the deleterious histological and ultrastructural changes induced by chronic AFB1 intake in rat SMSGs.

## Introduction

 Aflatoxins are naturally-occurring mycotoxins produced by the fungi *Aspergillus flavus*, a mold that can be found on several food products. They are probably the most popular and most intensively studied mycotoxins in the world ^1^. Aflatoxins could contaminate major agricultural commodities like corn, wheat, rice, barley, maize, peanut, onions and many other crops. They are also found in the milk, eggs and meat of animals that feed on aflatoxin-contaminated food ^2, 3^. Aflatoxins are highly toxic and could cause both acute and chronic toxicity in human beings and animals ^4^.

 Of the 20 types, Aflatoxin B1 (AFB1) is considered to be the most toxic. AFB1 toxicity was reported to lead to severe health issues such as cancer, growth retardation, and immunosuppression. In this regard, the International Agency for Research on Cancer classified AFB1 as a group I carcinogen, which raises the risk of cancer in humans ^5^. The oxidative stress caused by AFB1 is considered the main mechanism of action for AFB1-induced cell DNA, protein and lipid damage. Many studies suggested that toxicity may occur from the production of reactive oxygen species (ROS) intracellularly during AFB1 metabolism in the liver ^6, 7^.

 Antioxidants act as ‘free radical scavengers’, which avert and repair the damage caused by ROS. They stabilize or deactivate free radicals before the latter attack cells and biological targets ^8^. The scientific interest in the protective effect of antioxidants against aflatoxins has markedly increased in the last years ^9^. Among the plants recognized to present a rich source of antioxidants is *Rosmarinus officinalis* (common name, rosemary). Rosemary extract has also been shown to inhibit the fungal growth of *Asperigillus parasiticus* and subsequent aflatoxin production ^10^. In several studies rosemary has shown positive biological actions in terms of antioxidant activity, which reduced the harmful effects of AFB1 ^11– 13^.

 Information on the effect of AFB1 on the SMSG structure has been insufficient. Therefore, the aim of the present study was to examine the effect of AFB1 on the SMSG of albino rats using light and electron microscopes as well as explore the potential therapeutic effect of rosemary extract on aflatoxin-induced changes. Rats were used in this study because they have biology similar to humans and therefore can be a model for human toxicity.

## Methods

### Ethical statement

The study protocol and the procedures for animal care and experiments were approved by the Research Ethics Committee of the Faculty of Dentistry, Ain-Shams University, Egypt (#615).

All efforts were made to ameliorate any suffering of the animals by adopting the OECD 423 test guidelines.

### Animals

This study used 21 adult male albino rats, three months old and weighing 200-220 g. The animals were obtained from and housed in the Animal house, Faculty of Medicine, Ain Shams University. Sample size calculation was performed using G*Power version 3.1.9.2 (University Kiel, Germany) ^14^. The effect size was 0.95 using α level of 0.05 and β level of 0.05, i.e., power = 95%; the estimated sample size (n) was a total of 21 samples for three groups.

Rats were housed separately in cages (Suzhou Suhang Technology Equipment Co., Ltd.) in a constant temperature (22–24°C) and light-controlled room on an alternating 12:12 hour light-dark cycle and had free access to food and water. The animals were fed a natural diet and water ad libitum throughout the whole experiment.

All 21 rats were given a number (1–21) using a marker pen, then randomized by putting the numbers in an envelope and dividing them into three groups according to the numbers which were taken from the envelope

The three groups were as follows :

- Group C (saline-treated control group): rats were injected with 9% saline intraperitoneally once daily for four weeks.
- Group A (AFB1 treated group): rats were injected with AFB1 (Sigma Chemical Co., St. Louis, MO, USA) intraperitoneally with a dose of 2 mg/kg once daily for four weeks ^15^.
- Group R (rosemary treated group): rats were injected with AFB1 intraperitoneally using the same dose and period as in Group A followed by intraperitoneal injection of *Rosmarinus officinalis* extract (400mg/kg) for two weeks ^16^.

The rats were injected every morning at 9 am in the animal house laboratory of Ain Shams university.

### Rosemary methanolic extract

 Dried rosemary leaves were obtained from Harraz for food industry, natural products and botanical herbs (Product number 215). Dried rosemary leaves were ground for methanolic extraction, which was done as described by Hendel *et al.* ^17^. The obtained rosemary leaves were powdered and 100 g were submitted to hydrodistillation for 3 h with 1000ml distilled water using Clevenger type apparatus. The extracted oil was collected and dried over anhydrous sodium sulfate, then stored in sealed glass vials in a refrigerator at 4°C until use. From the plant extract material, 30 g was soaked for eight hours in methanol solvent at 40°C in a sterile conical flask. The mixture was then filtered using Whatman No. 4 filter paper to yield a light-brown filtrate. The methanolic filtrate was concentrated using a vacuum rotary evaporator (Heidolph 36001270 Hei-Vap Precision Rotary Evaporator) at 100 rpm for 30 minutes. The remaining extract was then dried in a vacuum oven for two hours to ensure the elimination of any residual solvent. The final extract was a dark green powder, which was then dissolved in 9% saline.

### Sample preparation and microscopy

 At the end of each experimental period, rats were euthanized by CO2 asphyxiation followed by cervical dislocation and their SMSGs were cut out performed by experienced animal laboratory personnal. SMSGs of the right side were fixed in neutral buffered formalin (10%), embedded in paraffin and sections 6 microns thick were sliced and stained with hematoxylin and eosin (H&E) for light microscopic examination using a Leica DM 1000 light microscope and camera and Leica Application suite-LAS software in the research center, The British University in Egypt. The specimens of the left sides were used for ultrastructural examination where small specimens were cut and fixed in 3% glutaraldehyde and osmium tetraoxide, dehydrated in alcohol and embedded in an epoxy resin. Microtome sections were prepared at approximately 500–1000 um thickness with a Leica Ultracut UCT ultramicrotome. Thin sections were stained with toluidine blue (1X), then sections were examined using a Lica ICC50 HD camera. Ultra-thin sections were prepared at approximately 75–90 um thickness and were stained with uranyl acetate and lead citrate, then examined using a JEOL (JEM-1400 TEM) transmission electron microscope.

## Results

### Light microscopic results

**Figure 1.**
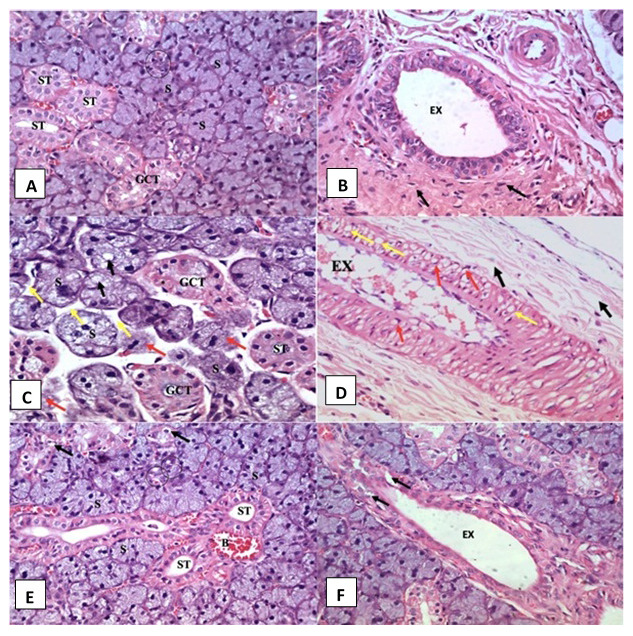
Photomicrographs of haemotoxylin and eosin (H&E) stained sections in submandibular salivary glands. ( **A**, **B**) Control group showing regular gland architecture of serous acini (S) intercalated duct (circle), granular convoluted tubule (GCT), striated duct (ST) and excretory duct (EX) surrounded by fibrous connective tissue stroma (arrows) (H&E orig. mag x 400). ( **C**, **D**) AFB1 treated group showing remnants of degenerated acini (red arrows), atrophic serous acini (S) with cytoplasmic vacuolation (black arrows), wide interacinar spaces (yellow arrows), vacuolated GCT and striated duct (ST) and excretory duct (EX) with thickened lining epithelium presenting extensive vacuolations (yellow arrows) and some crescent shaped nuclei in its cells (red arrows). Degenerated areas are seen in the surrounding fibrous connective tissue stroma (black arrows) (H&E orig. mag x 400). ( **E**, **F**) Rosemary treated group showing normal serous acini (S), intercalated ducts (circle), GCT with few cytoplasmic vacuoles (arrows) and striated ducts with normal cell lining (ST). Dilated and congested blood vessels were observed (B) and excretory duct (EX) shows relatively normal lining with slight vacuolations in some areas (arrow) (H&E orig. mag x 400).

Histological examination of SMSG of the control group is shown in Figure 1A and Figure 1B, which revealed normal histological features of the submandibular salivary gland.

Histological sections of SMSG of the AFB1 administered group rats showed acini apparently decreased in size, as revealed by wide interacinar spaces, and numerous different sized cytoplasmic vacuoles were seen in their cells ( Figure 1C). Striated ducts showed a decrease in size and loss of basal striations in the lining cells was observed. The presence of abundant cytoplasmic vacuolations were frequently observed in both striated and granular convoluted tubule (GCT) cells. Excretory duct cells showed extensive intracytoplasmic vacuolations and crescent-shaped nuclei. Retained secretory material was frequently found inside its lumen ( Figure 1D). Degeneration in the surrounding connective tissue fibers was also noted ( Figure 1D).

Histological examination of the SMSGs of the rosemary group rats presented almost identical histological features to the control group except with a few degenerative changes in both the acini and the ducts in some areas where few small intra-cytoplasmic vacuoles were detected. A limited number of congested and dilated blood vessels were observed close to striated ducts ( Figure 1D). Some lining cells of the excretory duct exhibited vacuolations in certain areas ( Figure 1F).

### Electron microscopic results

**Figure 2.**
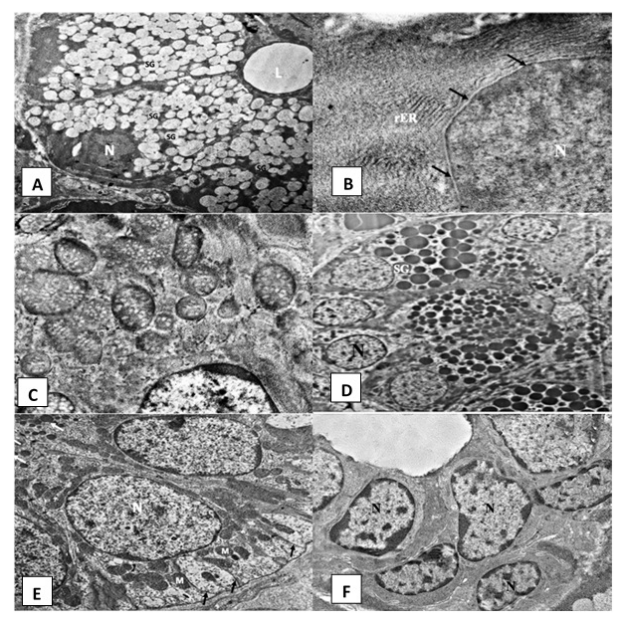
Electromicrographs of the control group showing ( **A**) acinar cells surrounding acinar lumen (L), rounded basally situated nuclei (N) and many secretory granules (SG) (uranyl acetate and lead x 5000). ( **B**) Part of an acinar open faced nucleus (N) with regular nuclear membrane (arrow) and parallel arrays of rough endoplasmic reticulum (rER) (uranyl acetate and lead x 12000). ( **C**) Normal acinar mitochondria with cristae (uranyl acetate and lead x 12000). ( **D**) Granular convoluted tubule with columnar cells and numerous electron dense secretory granules (SG) and basally situated open faced nucleus (N) (uranyl acetate and lead x 2500). ( **E**) Striated duct cells with open face nucleus (N), numerous basal infoldings (arrows) and radially arranged mitochondria (M) (uranyl acetate and lead x 5000). ( **F**) Excretory duct showing pseudostratified epithelium containing open face nucleus (N) (uranyl acetate and lead x 2500).

***Control group***. Ultrastructural examination of the SMSG of group A rats showed normal serous acini that were spherical in shape containing basally situated rounded nuclei (open-faced nucleus) and a narrow central lumen ( Figure 2A). Acinar cells exhibited parallel arrays of rough endoplasmic reticulum (rER) ( Figure 2B) and oval-shaped mitochondria ( Figure 2C). Secretory granules of variable sizes and different electron densities occupied most of the cytoplasm ( Figure 2A). The nuclei of the serous cells appeared euchromatic with a smooth, regular nuclear membrane ( Figure 2B). The GCTs presented long columnar cell lining. Their nuclei were large, rounded and basally located. Their cells contained numerous well-circumscribed membrane-bounded electron-dense secretory granules ( Figure 2D). The intralobular striated ducts consisted of long columnar cells. The basal cell membrane of the lining cells showed complex extensive infoldings. Few vertically arranged mitochondria were found interspersed with the folded membranes ( Figure 2E). The excretory duct was lined by pseudostratified epithelium containing open face nuclei ( Figure 2F).

**Figure 3.**
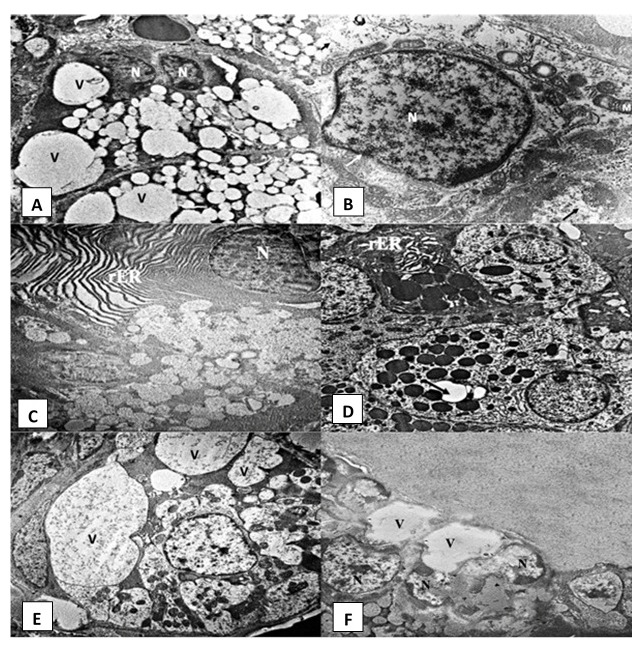
Electromicrographs of the AFB1 group showing ( **A**) serous acinus with different sized intracytoplasmic vacuolations (V) and shrunk nuclei (N) (uranyl acetate and lead x 5000). ( **B**) Open face nucleus of serous acinar cell (N) with irregular nuclear membrane (white arrows). Radially arranged mitochondria (M) with dilated cristae and vacuoles. Degenerated cytoplasm (black arrows) can be noticed (uranyl acetate and lead x 12000). ( **C**) Serous cell with irregular nuclear membrane (N) & dilated rough endoplasmic reticulum (rER) (uranyl acetate and lead x 5000). ( **D**) Granular convoluted tubule cells with intracytoplasmic vacuolations (arrows) and dilated rER (uranyl acetate and lead x 4000) ( **E**) Striated duct with indistinct cell outlines and numerous large intracytoplasmic vacuolations (V) pressing on the cell nucleus and loss of basal striations in some cells (uranyl acetate and lead x 5000). ( **F**) Excretory duct cells showing cytoplasmic vacuolations (V) and nuclei (N) with irregular outline (uranyl acetate and lead x 2500).

***AFB1 group***. Numerous large vacuolations were observed in the cytoplasm of acinar cells of AFB1 administered rats ( Figure 3A). Their nuclei showed irregular outline and clumping of chromatin material and exhibited differences in their shape and size, with variable degrees of shrinkage and pyknosis ( Figure 3A, Figure 3B and Figure 3C). rER presented luminal dilatation, discontinuity and fragmentation ( Figure 3C). Some acinar cells showed mitochondria with loss of cristae and the presence of vacuoles ( Figure 3B). The GCTs showed areas of degeneration in the form of a decrease in the number of electron-dense granules, intracytoplasmic vacuolations and dilatation in the rER ( Figure 3D). The irregular shaped cells of the striated duct revealed signs of degeneration displayed as shrunken nuclei and irregular nuclear outline. Degenerated organelles (especially dilated rER) and intra-cytoplasmic vacuolations were frequently encountered in the duct cells. Loss of basal infoldings on the striated ducts was also noticed ( Figure 3E). The excretory duct cells showed cytoplasmic vacuolations and nuclei with an irregular outline ( Figure 3F).

**Figure 4.**
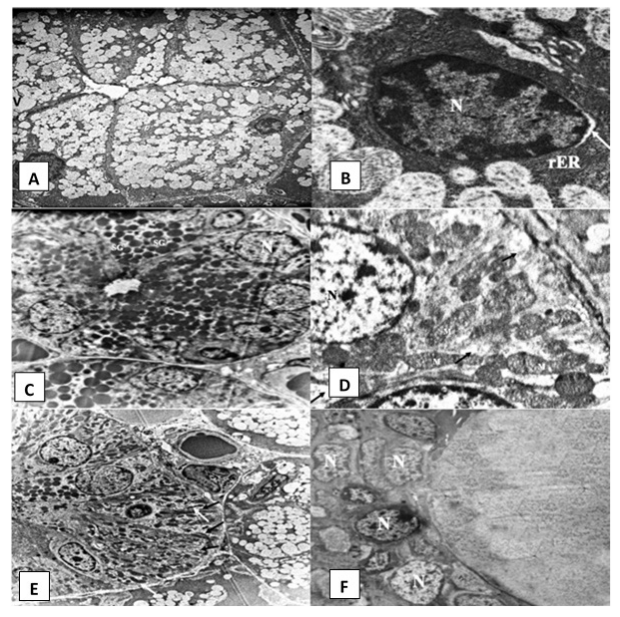
Electromicrographs of the rosemary treated group showing ( **A**) serous acinus containing nucleus (N), numerous secretory granules and few cytoplasmic vacuoles (uranyl acetate and lead x 5000). ( **B**) Open face nucleus (N) of an acinar cell and parallel arrays of rough endoplasmic reticulum (rER) with slight dilatations in some areas. Slight perinuclear space can be noticed (arrow) (uranyl acetate and lead x 12000). ( **C**) Granular convoluted tubule (GCT) containing basally situated nuclei (N) and numerous electron dense secretory granules (SG) (uranyl acetate and lead x 2500). ( **D**) Striated duct cell with open face nucleus (N) and tubular shaped mitochondria (M). Minimal cytoplasmic degeneration and some loss of basal infoldings can be observed (black arrows) (uranyl acetate and lead x 12000) ( **E**) Striated duct columnar cells with basal infoldings (black arrows) and numerous rod shaped mitochondria (white arrows) (uranyl acetate and lead x 5000). ( **F**) Excretory duct with pseudostratified epithelium lining cells presenting open face nucleus (N) (uranyl acetate and lead x 2500).

***Rosemary treated group***. Electron microscopic examination of SMSG of rats after treatment with rosemary revealed that the majority of acinar cells almost regained their normal architecture ( Figure 4A). Slightly dilated rER and very few cytoplasmic vacuolations were observed ( Figure 4B). GCTs were lined by long columnar cells with basally situated nuclei and many electron-dense secretory granules ( Figure 4C). Striated ducts regained their normal histological appearance, with the columnar cells having numerous basally located radially arranged rod-shaped mitochondria with minimal changes. Desmosomal junctions appeared in a normal pattern in between cells ( Figure 4D and Figure 4E). The excretory duct was lined by pseudostratified epithelium containing open face nuclei ( Figure 4F).

Original microscopy light and electron microscopy images for all groups are provided as *Underlying data* ^18^.

## Discussion

In poor developing countries, chronic intake of foods contaminated with aflatoxins is a prevailing problem in both humans and animals worldwide. Aflatoxins are considered of great global concern due to their carcinogenic and toxic effects on human and animal health ^4^. In the present study, AFB1 was administrated to the experimental rats for four weeks to simulate enough chronic exposure to cause oxidative stress in vital organs ^19^. The rats were administered the toxin intraperitoneally to ensure the delivery of the full dose to each animal.

Based on its popular use as an antioxidant in several studies on various tissues ^13, 20^, rosemary was chosen in this study to evaluate its potential for alleviating the histological and ultrastructural changes in the SMSG of aflatoxicated rats. Moreover, rosemary extracts have been found to have a positive effect on the natural antioxidants produced in the body. According to De Oliveira *et al.* ^11^, rosemary improves the production of endogenous antioxidants, leading to enhancement of the body’s antioxidant capacity, reduction in the levels of lipid peroxidation, and maintenance of cell membrane permeability. Furthermore, rosemary was chosen in the current study rather than synthetic antioxidants based on the study by Mira-Sánchez *et al.* ^21^, who found that the effect of rosemary compounds as an antioxidant was greater than the effect reported by artificial antioxidants.

In the present study, rosemary extract was administered at 400 mg/kg once daily for 14 days as Rahbardar *et al.* ^16^ documented that treatment of albino rats with rosemary extract with this dose and duration was enough to elicit its therapeutic potential.

The present results showed that AFB1 administration caused degenerative histopathological alterations in the SMSG of albino rats in the form of shrunken acini and numerous cytoplasmic vacuolations in acini, GCTs and excretory ducts. These results were in agreement with the study of Abou-Zeid *et al.* ^22^, who reported that SMSGs of rats exposed to AFB1 showed severe vacuolar degeneration in acinar cells and the epithelial cells of convoluted ducts. Moreover, the damaging effects of AFB1 detected in this study were also in parallel with other histopathological studies in which aflatoxins caused degeneration and numerous cytoplasmic vacuolations in hepatocytes of the liver and Langerhans cells of the pancreas of rats. The investigators attributed this to ROS production from the ingested aflatoxins ^23, 24^.

Coppes *et al.* ^25^ attributed the presence of acinar intracytoplasmic vacuoles to intra-cytoplasmic degenerative changes caused by the presence of proteolytic enzymes in their secretory granules which might infiltrate and cause damage to the cytoplasm, leading to autolysis and cellular death. However, Samah *et al.* ^26^ suggested that cytoplasmic vacuolations seen in acinar, GCT and excretory duct cells might be due to the ROS formation, resulting in lipid peroxidation and damage of cell membranes as well as membranes of the cell organelles, ending in impairment of the energy-dependent Na+ K+ ion pumps in the cell. These changes lead to accumulation of Na+ inside the cells with entry of water resulting in cellular swelling and vacuolations. In the present study, histological examination of the striated and excretory ducts showed dilatation of their lumens with retained secretion following AFB1 administration. Mahmoud *et al.* ^27^ attributed this finding to the accumulation of the salivary secretion and disturbance of exocytosis caused by glandular dysfunction. The changes in the connective septa reported in this study were inconsistent with several studies reporting that exposure to excessive amounts of toxins caused fibrosis and subsequently, overproduction of mature collagen fibrils ^22, 28^.

In this study AFB1 induced many alterations in SMSG tissue when observed under the electron microscope. AFB1 caused many signs of degeneration in the nuclei of the acinar cells manifested as an irregular outline and clumping of chromatin material in addition to changes in shape and size. In a study conducted to explore the apoptotic mechanism of AFB1, Mughal *et al.* ^29^ attributed shrunken nuclei and dense chromatin to increased expression of many proapoptotic proteins. Subsequently, DNA fragmentation and chromatin condensation results.

Yasuno *et al.* ^30^ suggested that the presented luminal dilatation of rER following AFB1 administration could be attributed to impaired secretory mechanisms and subsequently, the proteinaceous secretory material becomes concentrated within the arrays of the rER, which leads to their dilatation. Moreover, the degenerated mitochondria in the cells of the acini and ducts in the aflatoxin-administered group in this study might be due to ROS production and/or to the preferential binding of aflatoxins to mitochondrial DNA, which hinders ATP production, causing disruption of mitochondrial functions and mitochondrial membranes ^31^.

This research revealed that rosemary extract ameliorated the damaging effects of AFB1 on SMSG, which was observed histologically and ultrastructurally, where most alterations caused by AFB1 administration were greatly reduced. The present results might be explained by Ghoneim & Arafat ^32^ who reported that treatment with rosemary extract prevented oxidative stress caused by ROS on rat parotid salivary glands. They attributed this improvement to the antioxidative properties of rosemary, which resulted in the subsidence of oxidative stress. Moreover, De Oliveira *et al.* ^33^ attributed the improvement in the mitochondrial structure observed upon treatment with rosemary to the effect of carnosic acid, which avoided mitochondrial membrane potential disturbance and reduced the levels of oxidative stress markers in mitochondrial membranes.

From the present study, it could be concluded that chronic intake of AFB1 gave rise to deleterious histological and ultrastructural changes in the SMSGs of albino rats and that treatment with rosemary extract was able to ameliorate this effect.

## Data availability

### Underlying data

Figshare: Effects of aflatoxin B1 on the submandibular salivary gland of albino rats and possible therapeutic potential of Rosmarinus officinalis: a light and electron microscopic study. https://doi.org/10.6084/m9.figshare.12644855.v2 ^18^

This project contains the following underlying data:

- Original, unedited electron microscopy images in JPG format (em1.jpg - em18.jpg)
- Original, unedited light microscopy images in TIF format (lm1.tif - lm6.tif)

Data are available under the terms of the Creative Commons Zero "No rights reserved" data waiver (CC0 1.0 Public domain dedication).
