# Supplementary material for: Effects of aflatoxin B1 on the submandibular salivary gland of albino rats and possible therapeutic potential of Rosmarinus officinalis: a light and electron microscopic study
Source: F1000Res. 2020 Jul 21;9:752. [Version 1] doi: 10.12688/f1000research.25196.1 (PMC7388195; doi:10.12688/f1000research.25196.1)
Supplement: Peer review history

# Peer review: Reviewer response for version 1

**DOI:** 10.5256/f1000research.27805.r67567

**Published:** 2020-07-28

**Authors:** Dina Rady

**Affiliations:** 1 Oral Biology Department, Faculty of Dentistry, Cairo University, Cairo, Egypt

The idea of the research article titled: “Effects of aflatoxin B1 on the submandibular salivary gland of albino rats and possible therapeutic potential of Rosmarinus officinalis: a light and electron microscopic study” is very interesting and valuable as the researcher illustrated the effect of AFB1 on the submandibular salivary glands of albino rats. Besides, the authors  investigated the possible therapeutic effect of *Rosmarinus officinalis* extract which could be a base for clinical application.

 The article is clear and easy to comprehend as it is well constructed. Significant results were noted in the results of this study, all the photomicrographs are very clear. But still, there are suggested minor comments the authors could deal with, or at least discuss for additional impact. 

In the methodology:

1. The letters used to identify different groups can be changed into numbers to be easily identified.
2. It is an idea to perform an additional assessment method. Example: RT-PCR analysis of α-amylase gene expression to confirm the obtained results.

Is the work clearly and accurately presented and does it cite the current literature?

Yes

If applicable, is the statistical analysis and its interpretation appropriate?

Yes

Are all the source data underlying the results available to ensure full reproducibility?

Yes

Is the study design appropriate and is the work technically sound?

Yes

Are the conclusions drawn adequately supported by the results?

Yes

Are sufficient details of methods and analysis provided to allow replication by others?

Yes

Reviewer Expertise:

Oral Biology

I confirm that I have read this submission and believe that I have an appropriate level of expertise to confirm that it is of an acceptable scientific standard.

# Peer review: Reviewer response for version 1

**DOI:** 10.5256/f1000research.27805.r67572

**Published:** 2020-07-22

**Authors:** Mahmoud Serag

**Affiliations:** 1 Division of Prosthodontics, Department of General Dentistry, School of Dental Medicine, East Carolina University, Greenville, NC, USA

The present article by Bassant *et al.* is well written and the results are well presented in the manuscript. The methods were clearly described and thorough details were provided which adds to the scientific value of the manuscript. Power analysis details are provided for the sample size calculation. Light microscopic and electron microscopic photos are clear and described sufficiently.

 However, I have some remarks that might add to the manuscript:

- The authors could add the use of an immunohistochemical marker for oxidative stress to confirm histological findings.
- Grouping could be renamed I, II and III instead of the current namings. 
- In the first group, normal saline is 0.9% not 9.

I believe this manuscript is suitable for publication.

Is the work clearly and accurately presented and does it cite the current literature?

Yes

If applicable, is the statistical analysis and its interpretation appropriate?

Not applicable

Are all the source data underlying the results available to ensure full reproducibility?

Yes

Is the study design appropriate and is the work technically sound?

Yes

Are the conclusions drawn adequately supported by the results?

Yes

Are sufficient details of methods and analysis provided to allow replication by others?

Yes

Reviewer Expertise:

Dentistry

I confirm that I have read this submission and believe that I have an appropriate level of expertise to confirm that it is of an acceptable scientific standard.
